# Development of an LC-MS Method for the Analysis of Birch (*Betula* sp.) Bark Bioactives Extracted with Biosolvents

**DOI:** 10.3390/molecules30153181

**Published:** 2025-07-29

**Authors:** Inmaculada Luque-Jurado, Jesús E. Quintanilla-López, Rosa Lebrón-Aguilar, Ana Cristina Soria, María Luz Sanz

**Affiliations:** 1 Instituto de Química Orgánica General (CSIC), Juan de la Cierva 3, 28006 Madrid, Spain; iluque@iqog.csic.es (I.L.-J.); acsoria@iqog.csic.es (A.C.S.); 2 Instituto de Química-Física ‘Blas Cabrera’ (CSIC), Serrano 119, 28006 Madrid, Spain; je.quintanilla@iqf.csic.es (J.E.Q.-L.); rlebron@iqf.csic.es (R.L.-A.)

**Keywords:** birch (*Betula* sp.) bark, bioactive triterpenoids, liquid chromatography-mass spectrometry (LC-MS), biosolvents, natural deep eutectic solvents (NADESs)

## Abstract

Birch (*Betula* sp.) bark is a well-known natural source of betulin (Bet) and betulinic acid (BAc), both of which have several bioactive properties. The evaluation of the extraction performance, relative to these lupane-type triterpenoids, provided by different biosolvents requires the development of a high-resolution and high-sensitivity liquid chromatography-mass spectrometry (LC-MS) approach that is also compatible with challenging extractants such as natural deep eutectic solvents (NADESs). In this work, an LC-MS method was developed and analytically characterized prior to its application for the quantitation of Bet and BAc in birch bark extracts obtained using conventional solvents (methanol and acetone) and biosolvents (limonene and NADESs). High precision (RSD < 3.3%), sensitivity (LOD: 23 ng mL^−1^ and 29 ng mL^−1^ for Bet and BAc, respectively), and accuracy (95–102% recovery) were found for this optimized method, using an acidulated water–methanol mixture as the mobile phase and sodium acetate as an additive. Extraction experiments conducted at 55 °C revealed that the NADESs, particularly thymol:1-octanol (1:1 molar ratio), outperformed the other solvents and were highly effective for the recovery of both triterpenoids (17.50 mg g^−1^ and 0.92 mg g^−1^ of Bet and BAc, respectively). This method can also be applied to similar extracts obtained from other biomasses.

## 1. Introduction

Birch (*Betula* sp.) bark (BB) is a natural source of valuable bioactive compounds, particularly betulin (Bet) and betulinic acid (BAc). These lupane-type triterpenoids have attracted great scientific interest due to their wide range of pharmacological properties and potential therapeutic applications [1]. Betulin, a naturally occurring pentacyclic triterpene, is abundantly present in the outer bark of birch trees, where it contributes to the protective functions of the plant. It is known for its anti-inflammatory, antiviral, antibacterial, and antioxidant activities, among others. Moreover, betulin serves as a precursor for the synthesis of various biologically active derivatives, including betulinic acid. This acid also exhibits enhanced pharmacological effects, such as antitumor, anti-inflammatory, and hepatoprotective properties [2,3].

BB bioactives are typically extracted via solid–liquid extraction (SLE). While conventional organic solvents such as dichloromethane, ethyl acetate, etc., have demonstrated varying degrees of efficiency in extracting both Bet and BAc [4,5,6,7], their use raises environmental and safety concerns due to toxicity, volatility, and non-renewability. To address these issues, increasing attention is being paid—in a limited number of studies—to the use of biosolvents as greener extractants. Thus, monoterpenes such as limonene and pinene have been reported to provide high yields in the microwave-assisted extraction of BB Bet [8,9]. More recently, natural deep eutectic solvents (NADESs) have been proven to be advantageous for the SLE of BAc and other triterpenic acids from *Eucalyptus globulus* biomass [10], though their application for the efficient and simultaneous recovery of Bet and BAc from BB has not yet been explored.

Comparing the performances of all these sustainable extraction procedures, as well as the characterization of the composition of BB extracts prior to their application, requires the availability of high-resolution, sensitive, and reliable analytical methods compatible with both the target bioactives and the extractants. However, this remains a challenging task, due to the issues that commonly arise when using NADESs, which are typically related to the chromatographic process and detection efficiency. While gas chromatography coupled to mass spectrometry (GC-MS) has successfully been applied for the determination of lupane-type triterpenoids in several vegetal matrices, including birch extracts [8], the low volatility of these analytes requires their previous derivatization, making this procedure long and laborious. Regarding liquid chromatography (LC), methods based on spectrophotometric detection have been shown to provide a low sensitivity; this is associated with the lack of highly UV-absorbing chromophores in pentacyclic triterpenoids [10,11,12]. Although a few procedures based on mass spectrometry (MS) detection, and featuring improved analytical properties, have been reported [11,13,14,15], none of them have addressed the quantitation of these bioactives in BB extracts, a particularly challenging task when NADESs are used as the extractants.

Due to their high viscosity, NADESs can hinder sample injection and reduce reproducibility. NADES components, when dissociated in the mobile phase, can interfere with detection by co-eluting with target compounds or inducing ion suppression or enhancement in MS detection. An evaluation of their miscibility in LC mobile phases should also be performed to avoid phase separation, baseline instability, and peak distortion [16,17,18,19]. Therefore, the ever-essential analytical characterization of LC-MS methods intended for the quantitation of BB bioactives should additionally include a consideration of the singularities referred to above when dealing with NADES extracts; this presents an issue not previously addressed in the literature.

The aim of this work was the development of an LC-MS method for the quantitative determination of Bet and BAc in BB extracts obtained using biosolvents, particularly NADESs. The method was designed to address the specific analytical challenges posed by NADESs and was also applied to extracts obtained with conventional solvents (methanol, acetone) and monoterpenes (e.g., limonene) for comparative purposes. It was intended that the optimized LC-MS method would allow an accurate and reproducible quantitation of Bet and BAc across different solvent systems. Moreover, NADES-based extraction would offer improved recovery of these bioactives, thereby supporting greener strategies for BB valorization.

## 2. Results and Discussion

### 2.1. Development of an LC-MS Method for the Analysis of Bet and BAc

As previously mentioned, the optimization of an LC-MS method was required for its intended application for the evaluation of different green solvents as Bet and BAc extractants. Different binary mixtures consisting of H_2_O with a 0.1% formic acid (phase A) and either acetonitrile or methanol (phase B) as the mobile phase were evaluated. Acetonitrile mixtures providing a higher elution strength were first assayed under both gradient and isocratic conditions; however, they did not provide satisfactory baseline resolution of both Bet and BAc from other birch bark or NADES components. Considering that the choice of a proton-donor solvent such as methanol as a component of the mobile phase may favor ionization in the positive ion generation mode of Bet, as previously described in the literature [13], further optimization of the separation was performed using methanol as the organic phase. Among the different conditions assayed, isocratic separations using 85% methanol (v/v) were selected as a trade-off between the resolution and retention time (Bet: 13.7 min; BAc: 16.0 min) of BB bioactives, with no issues encountered regarding extractant immiscibility or interferences. No improvement in separation was observed when methanol with 0.1% formic acid was used instead of pure methanol.

**Figure 1:**
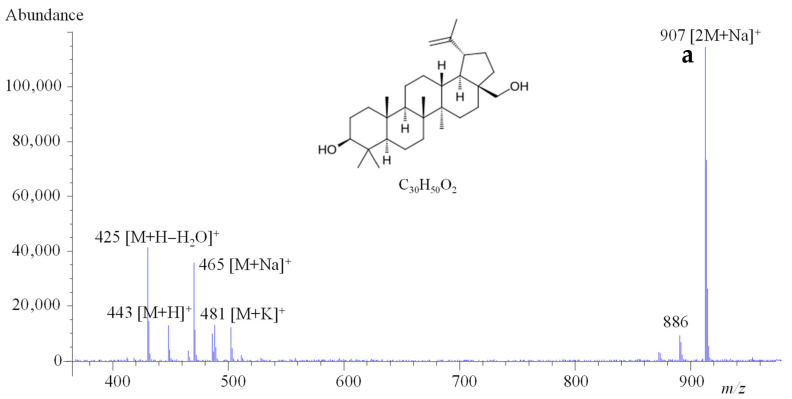

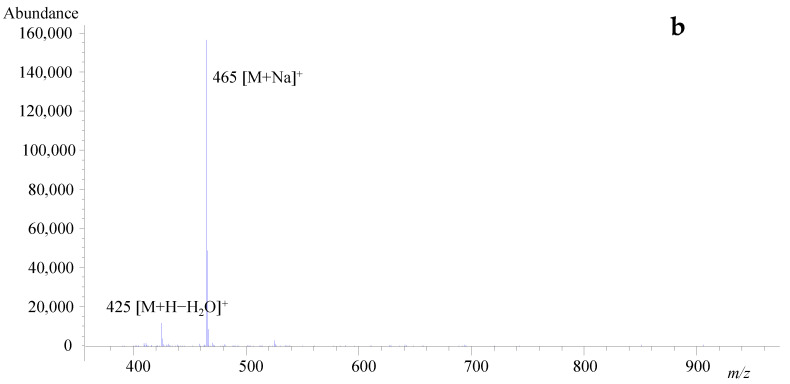
Chemical structure, molecular formula and ESI(+) mass spectra of Bet obtained using as mobile phase (**a**) 15% water with 0.1% formic acid: 85% methanol or (**b**) 14% water with 0.1% formic acid: 1% sodium acetate 1 mM: 85% methanol.

**Figure 2:**
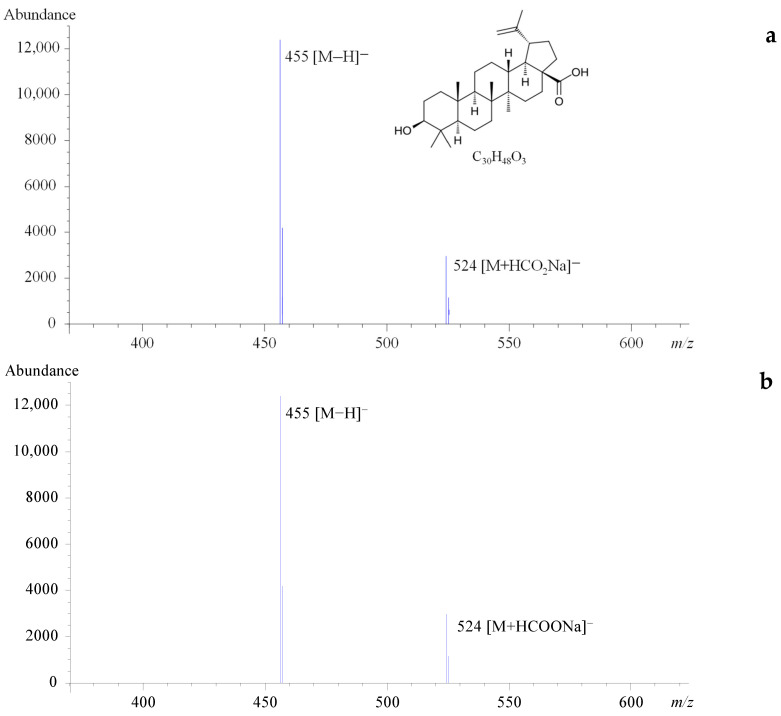
Chemical structure, molecular formula and ESI(−) mass spectra of BAc obtained using as mobile phase (**a**) 15% water with 0.1% formic acid: 85% methanol or (**b**) 14% water with 0.1% formic acid: 1% sodium acetate 1 mM: 85% methanol.

Regarding the optimization of ion transmission into the analyzer, betulin was better detected in positive mode, and the ions with the highest intensity were found to be *m*/*z* 907, corresponding to the sodiated adduct of the dimer ([2M+Na]^+^), followed by *m*/*z* 465 and *m*/*z* 425, corresponding to [M+Na]^+^ and [M+H−H_2_O]^+^, respectively. Signals of [M+H]^+^ and [M+K]^+^ and the protonated ion of the dimer [2M+H]^+^ were also detected (Figure 1a). As for the optimal response of BAc, the highest ion under negative polarity was *m*/*z* 455 ([M−H]^−^), followed by *m*/*z* 524 ([M+HCOOH+Na−H]^−^) and *m*/*z* 501 ([M+HCOOH−H]^−^) (Figure 2a). No differences in Bet and BAc adducts were observed with the extractant considered.

Whereas the intensity of the [M-H]^−^ ion for BAc was found to be stable over time, a variable abundance of [2M+Na]^+^, [M+Na]^+^, and [M+H−H_2_O]^+^ ions for Bet was observed, even when the LC-MS operating conditions were unchanged. As the acquisition in SIM mode using selected *m*/*z* ratios was chosen for the quantitation of both Bet and BAc, the stabilization of the Bet signal was evaluated by using sodium acetate as the mobile phase modifier. Using this mobile phase, different isocratic and gradient elution programs were assayed and the optimum separation and peak shape were obtained for 14% H_2_O + 0.1% formic acid (A)/1% sodium acetate 1 mM (B)/85% methanol (C) (0–25 min); 1% A/1% B/98% C (26–31 min); and reversion to initial conditions (32–41 min). Under these conditions, [M+Na]^+^ was stabilized as the highest-intensity ion for Bet (Figure 1b) and no significant changes were observed in the BAc spectrum (Figure 2b).

The MS conditions were also optimized to maximize Bet and BAc responses. Firstly, different capillary voltages (3000 and 4000 V) were tested. As shown in Figure S1, the highest response of both Bet and BAc was obtained at 4000 V, and therefore this value was selected for further experiments. The drying gas flow rate (6 and 12 L min^−1^; Figure S2) and temperature (300–350 °C; Figure S3) were also considered. While 12 L min^−1^ and 350 °C provided the highest intensities for Bet, only slight differences within the ranges assayed were observed for BAc. Therefore, these conditions were set as optimal for both compounds. As nebulizing gas pressures of 10, 35, and 55 psig yielded comparable results for both compounds (Figure S4), a pressure of 35 psig was selected as a mid-range value. Finally, different fragmentor voltages for both Bet and BAc were evaluated (Figure S5), as higher voltages are known to enhance ion transmission and signal intensity, but they may also promote fragmentation [20]. In the case of betulin, a slightly higher response was observed at 160 V compared to 130 and 100 V, whereas the highest response for BAc was achieved at 300 V.

As a result, the optimal ESI values providing the highest response for these stabilized signals were found to be capillary voltage, 4000 V; drying gas flow and temperature, 12 L min^−1^ and 350 °C; nebulizing gas pressure, 35 psig; and fragmentor voltage, 160 V for Bet and 300 V for BAc.

### 2.2. Analytical Characterization

Once the LC-MS method had been developed, its comprehensive analytical characterization for the analysis of Bet and BAc in BB extracts was addressed (Table 1).

First, the potential matrix effect associated with coextracted BB compounds or the solvent used as the extractant was evaluated. A 1:133 (*v*/*v*) dilution with methanol was found to be required to rule out this effect and to assure the reliable quantitation of target bioactives irrespective of the BB extract considered.

The linearity of the method in the concentration ranges encompassing the contents of Bet and BAc in the BB samples under study was found to be good, with coefficients of determination (R^2^) exceeding 0.994. In contrast to other LC-MS approaches [13,15], in which a three-orders-of-magnitude linearity was obtained for both bioactives, the linear response experimentally determined in the present study was found to be much more reduced for BAc (0.098–2 µg mL^−1^) as compared to Bet (0.075–100 µg mL^−1^). The intra-day and inter-day precision for target birch bark bioactives was good (relative standard deviations (RSD) ≤ 3%), and similar to the values in previous reports [5].

As expected for compounds sharing a lupane-type structure, a similar sensitivity for both target bioactives was obtained, with *LOD* and *LOQ* as low as 0.023 µg mL^−1^ and 0.075 µg mL^−1^ for Bet and 0.029 µg mL^−1^ and 0.098 µg mL^−1^ for BAc, respectively. Although a lower sensitivity for ESI has been reported in a comparative study on the evaluation of different MS interfaces for the LC-MS analysis of pentacyclic triterpenoids [15], the results here obtained using an ESI interface were similar to those provided by an atmospheric pressure photoionization (APPI) source (*LOQ*: 0.04 µg mL^−1^ for Bet and 0.07 µg mL^−1^ for BAc), with both configurations operating under SIM mode.

Recovery values greater than 95%, calculated by spiking the BB NADES3 extract with different amounts of standards, assured the intended accurate quantitation of both Bet and BAc in BB extracts.

### 2.3. Application of the Optimized LC-MS Method to Different BB Extracts

Once the optimized LC-MS method was proven to allow the resolution of the two target bioactives with similar structures (Bet and BAc) here considered, and also their reliable quantitation, its application to the evaluation of the extraction performance of different BB extractants was carried out.

Table 2 shows the concentrations of Bet and BAc extracted from BB with NADES1-4 at 25 °C and 55 °C. Conventional solvents (methanol and acetone) commonly used for the extraction of these bioactives [5] and limonene [9] were also included for comparative purposes.

As evidenced by the LC-MS method developed, at 25 °C, the highest concentrations of Bet and BAc were extracted using the conventional solvents acetone (14.22 mg g^−1^ and 0.85 mg g^−1^, respectively) and methanol (12.75 mg g^−1^ and 0.86 mg g^−1^, respectively), followed by NADES2 and NADES3. Despite the good results obtained by Grazhdannikov et al. [9] for the extraction of Bet from birch bark using limonene, this extractant provided the lowest yields of both triterpenoids (4.26 mg g^−1^ and 0.38 mg g^−1^, respectively) in the present study.

As expected, extracts enriched in target bioactives were obtained at temperatures above ambient temperature, irrespective of the solvent considered. Thus, at 55 °C, NADES3, similar to conventional solvents, gave rise to the highest extraction of Bet (17.5 mg g^−1^), whereas the lowest concentration of this compound was again extracted with limonene. Regarding BAc, all biosolvents (NADESs and limonene) provided significantly higher concentrations (0.93–0.63 mg g^−1^) than methanol (0.48 mg g^−1^) and acetone (0.47 mg g^−1^). These preliminary results highlight the efficiency of NADESs and limonene for the simultaneous extraction of high-value BB bioactives at 55 °C. Moreover, these solvents offer advantages in terms of environmental sustainability and safety over conventional ones [21].

## 3. Materials and Methods

### 3.1. Samples and Standards

Analytical standards of Bet and BAc (>99% purity) were acquired from Sigma-Aldrich (St. Louis, MO, USA). A birch bark (BB) sample of *Betula* sp. was purchased online (Farmasana, Zaragoza, Spain). This sample was ground to fine particles using an IKA A10 basic mill (IKA-Werke, Staufen, Germany), sieved through a 500 µm mesh, and stored in amber vials at room temperature until extraction.

### 3.2. NADES Preparation

Four different NADESs were prepared according to the method reported by Dai et al. [22]. Thymol was used as the hydrogen bond donor (HBD) in all cases, and different hydrogen bond acceptors (HBA) were considered, i.e., NADES1: octanoic acid; NADES 2: 1-propanol; NADES 3: 1-octanol; and NADES 4: menthol (all reagents were acquired from Sigma-Aldrich). These components were mixed in a 1:1 molar ratio and heated in a water bath at 60 °C for 1 h, under continuous magnetic stirring (550 rpm), until a clear and homogeneous liquid was obtained. NADESs were stored at room temperature, in the absence of light, prior to use. The stability of the prepared NADESs was assured for one week. Viscosity (mPa s) and density (g cm^−3^) data of the NADESs here prepared was previously reported by de la Puerta et al. [23].

### 3.3. Betulin and Betulinic Acid Extraction

The BB sample (100 mg) was mixed with 1 mL of the previously prepared NADESs, limonene (97% purity, Sigma-Aldrich), and conventional solvents [methanol (J.T. Baker, Phillipsburg, NJ, USA) and acetone (Scharlab, Barcelona, Spain)] and stirred at 600 rpm for 30 min at either 25 °C or 55 °C using a heating plate (RCT basic, IKA). Extracts were centrifuged at 4400× *g* for 10 min, diluted as required with methanol, and filtered through 0.2 µm polytetrafluoroethylene (PTFE) membranes (Interchim, Montluçon, France) prior to LC-MS analysis. All extractions were performed in triplicate.

### 3.4. LC-MS Analysis

LC-MS analyses were performed on a 1260 Infinity II Prime LC system provided with an autosampler, quaternary pump, column heater compartment, and diode array detector, and coupled via an electrospray ionization (ESI) source to a 6125 single quadrupole mass spectrometry detector (both from Agilent Technologies, Santa Clara, CA, USA). Separations were carried out using a Poroshell 120 EC-C18 column (150 × 3 mm, 2.7 μm; Agilent Technologies) thermostatized at 35 °C.

A number of binary mixtures/gradients consisting of Milli-Q water and acetonitrile (J.T. Baker) or methanol, including those prepared using different mobile phase modifiers [formic acid (Thermo Fisher Scientific, Waltham, MA, USA) and sodium acetate (Sigma-Aldrich)], were assayed for the resolution of target bioactives and for the stabilization of their MS responses. Injection volume and flow rate were set at 10 μL and 0.3 mL min^−1^, respectively.

The optimization of the ESI source was carried out by an infusion of Bet and BAc standard solutions in methanol (0.04 and 0.02 mg mL^−1^, respectively) at 10 µL min^−1^ under positive and negative polarities. Different values for the fragmentor voltage (100–300 V), nebulizing gas (N_2_, 99.5% purity), pressure (10, 35 and 55 psig), capillary voltage (3000 and 4000 V), drying gas (N_2_, 99.5% purity), flow (6 and 12 L min^−1^), and temperature (325 and 350 °C) were considered to maximize the responses of these two bioactives.

Compound identification was based on chromatographic retention data and full-scan mass spectra (100–1000 *m*/*z* range) and confirmed by co-injection of the corresponding commercial standards. The quantitation (*n* = 3) of BB bioactives, after the dilution of extracts in methanol as required, was performed by using the external standard method, employing data acquired in single ion monitoring (SIM) mode for selected adduct ions (*m*/*z* 465 for Bet and *m*/*z* 455 for BAc). Standard solutions in the ranges 0.0002–0.1 mg mL^−1^ and 0.0001–0.1 mg mL^−1^ were prepared for the Bet and BAc calibration curves, respectively. Data acquisition and processing were performed using OpenLAB CDS Software (v.2.19.20, Agilent Technologies). The results are expressed as mg g^−1^ of sample.

The LC-MS method previously optimized for the quantitation of BB bioactives was further analytically characterized. First, an evaluation of the possible matrix effect was performed through an analysis of a series of dilutions (in the range 1:80–1:2560, *v*/*v*) of BB NADES extracts. Reproducibility was evaluated in terms of intra- and inter-day precision by analysis (*n* = 5) within the same day or in five consecutive days, respectively, of Bet and BAc standard solutions in NADES3, diluted as required with methanol (1:133, *v*:*v*) to achieve concentrations of 0.03 mg mL^−1^ of Bet and 0.002 mg mL^−1^ of BAc. The linearity of Bet and BAc responses over the expected concentration range of these bioactives in the samples under study was determined. Goodness of fit for these calibration curves was assessed using the corresponding coefficients of determination (R^2^). Limits of detection (*LOD*) and quantitation (*LOQ*) were calculated as three and ten times the signal-to-noise ratio (*S/N*), respectively. The accuracy of quantitation was estimated by spiking BB extracts (*n* = 3) with different amounts of target bioactives (200–400 µL of Bet 0.03 mg mL^−1^, 400–800 µL of BAc 0.002 mg mL^−1^), and further calculation of recovery (%).

### 3.5. Statistical Analysis

Statgraphics Centurion XVI software (Statgraphics Technologies, Inc., The Plains, VA, USA) was used for statistical analysis. The significance (*p* < 0.05) of differences among the extractions obtained using different extractants or under different extraction temperatures was determined by analysis of variance (ANOVA, Tukey test).

## 4. Conclusions

An improved LC-MS method was developed for the quantitative determination of Bet and BAc. Although a narrower linear range was found for BAc in comparison to that for Bet, the good analytical performance observed in terms of reproducibility, efficiency, and sensitivity enabled its use for the accurate and reliable quantitation of both high-value bioactives when present in BB extracts of different natures, as required in studies aimed at extractant optimization. Special emphasis has also been placed on the evaluation of the applicability of this approach to NADES extracts, as they may affect the qualitative and quantitative performance of the optimized LC-MS method to a greater extent.

Regarding extraction, the results obtained at 55 °C demonstrated that, among the NADESs evaluated, NADES3 (thymol:1-octanol) was a highly effective extractant of BB bioactives, particularly when the simultaneous recovery of both Bet and BAc is to be maximized.

Finally, the versatility of the LC-MS methodology described herein allows its use for several applications in different fields, such as in nutraceutical standardization or quality control of green BB extracts, in which the analysis of these lupane-type triterpenoids is intended. The application of this method to other types of biomass would require a specific evaluation of its analytical parameters in order to ensure its robustness and applicability.

## Figures and Tables

**Table 1:** Analytical characterization of the LC-MS method developed for analysis of BB bioactives.

	Bet	BAc
Calibration curve	y = 2.88·10^8^ x − 13.754 R^2^ = 0.994	y = 2.40·10^8^ x + 26.317 R^2^ = 0.996
Linear range (µg mL^−1^)	0.075–100	0.098–2
Intraday precision (RSD%, *n* = 5)	0.52	0.61
Inter-day precision (RSD%, *n* = 5)	3.04	3.28
*LOD* (µg mL^−1^)	0.023 (0.0001) *	0.029 (0.00004)
*LOQ* (µg mL^−1^)	0.075 (0.0003)	0.098 (0.0002)
Accuracy (%)	102.27 (2.10)	94.76 (2.13)

* Standard deviation in parenthesis for *n* = 3.

**Table 2:** Concentration (mg g^−1^) of betulin (Bet) and betulinic acid (BAc) extracted from birch bark using different solvents and temperatures.

	25 °C		55 °C	
Extractant	Bet	BAc	Bet	BAc
NADES1 (thymol:octanoic acid)	8.42 (0.73) *^d^	0.65 (0.04) ^c^	14.38 (0.59) ^b^	0.91 (0.03) ^a^
NADES2 (thymol:1-propanol)	10.84 (0.21) ^c^	0.75 (0.01) ^b^	12.66 (0.50) ^bc^	0.88 (0.06) ^a^
NADES3 (thymol:1-octanol)	9.81 (0.69) ^c^	0.67 (0.02) ^c^	17.50 (1.67) ^a^	0.92 (0.04) ^a^
NADES4 (thymol:menthol)	7.56 (0.05) ^d^	0.600 (0.004) ^c^	14.31 (0.51) ^b^	0.93 (0.02) ^a^
Limonene	4.26 (0.01) ^e^	0.38 (0.02) ^d^	11.28 (0.33) ^c^	0.63 (0.04) ^b^
Methanol	12.75 (0.79) ^b^	0.86 (0.04) ^a^	17.66 (0.47) ^a^	0.48 (0.01) ^c^
Acetone	14.22 (0.21) ^a^	0.85 (0.05) ^a^	17.61 (0.21) ^a^	0.47 (0.01) ^c^

^a–e^ Different letters indicate significant differences in concentration (*p* < 0.05) for the solvents evaluated under the selected temperatures. * Mean concentration and standard deviation values for *n* = 3.

## Data Availability

Data is available upon request to interested researchers.

## Supplementary Materials

The following supporting information can be downloaded at: https://www.mdpi.com/article/10.3390/molecules30153181/s1, Figure S1. Effect of capillary voltage (V) on the ESI-MS signal intensity of (A) betulin ([M+H]^+^ = 465) and (B) betulinic acid ([M−H]^−^ = 455). Figure S2: Effect of drying gas flow rate (L min^−1^) on the ESI-MS signal intensity of (A) betulin ([M+H]^+^ = 465) and (B) betulinic acid ([M−H]^−^ = 455). Figure S3: Effect of drying gas temperature (°C) on the ESI-MS signal intensity of (A) betulin ([M+H]^+^ = 465) and (B) betulinic acid ([M−H]^−^ = 455). Figure S4: Effect of nebulizing gas pressure (psig) on the ESI-MS signal intensity of (A) betulin ([M+H]^+^ = 465) and (B) betulinic acid ([M−H]^−^ = 455). Figure S5: Effect of fragmentor voltage (V) on the ESI-MS signal intensity of (A) betulin ([M+H]^+^ = 465) and (B) betulinic acid ([M−H]^−^ = 455).
