# Supplementary material for: Development of an LC-MS Method for the Analysis of Birch (Betula sp.) Bark Bioactives Extracted with Biosolvents
Source: Molecules. 2025 Jul 29;30(15):3181. doi: 10.3390/molecules30153181 (PMC12348499; doi:10.3390/molecules30153181)
Supplement: Supplementary file 1 [file molecules-30-03181-s001.zip › molecules-3760380-supplementary.pdf]

Supplementary Materials for

Development of an LC-MS Method for the Analysis of Birch (*Betula* sp.) Bark Bioactives Extracted with Biosolvents

Inmaculada Luque-Jurado [1], Jesús E. Quintanilla-López [2], Rosa Lebrón-Aguilar [2], Ana Cristina Soria [1] and María Luz Sanz [1,*]

[1] Instituto de Química Orgánica General (CSIC), Juan de la Cierva 3, 28006 Madrid, Spain; iluque@iqog.csic.es (I.L.-J.); acsoria@iqog.csic.es (A.C.S.)
[2] Instituto de Química-Física 'Blas Cabrera' (CSIC), Serrano 119, 28006 Madrid, Spain; je.quintanilla@iqf.csic.es (J.E.Q.-L.); rlebron@iqf.csic.es (R.L.-A.)
* Correspondence: mlsanz@iqog.csic.es

Table of contents:

Figure S1. Effect of capillary voltage (V) on the ESI-MS signal intensity of (A) betulin ($[M+H]^+ = 465$) and (B) betulinic acid ($[M-H]^- = 455$).

Figure S2: Effect of drying gas flow rate (L min^{-1}) on the ESI-MS signal intensity of (A) betulin ($[M+H]^+ = 465$) and (B) betulinic acid ($[M-H]^- = 455$).

Figure S3: Effect of drying gas temperature (º C) on the ESI-MS signal intensity of (A) betulin ($[M+H]^+ = 465$) and (B) betulinic acid ($[M-H]^- = 455$).

Figure S4: Effect of nebulizing gas pressure (psig) on the ESI-MS signal intensity of (A) betulin ($[M+H]^+ = 465$) and (B) betulinic acid ($[M-H]^- = 455$).

Figure S5: Effect of fragmentor voltage (V) on the ESI-MS signal intensity of (A) betulin ($[M+H]^+ = 465$) and (B) betulinic acid ($[M-H]^- = 455$).

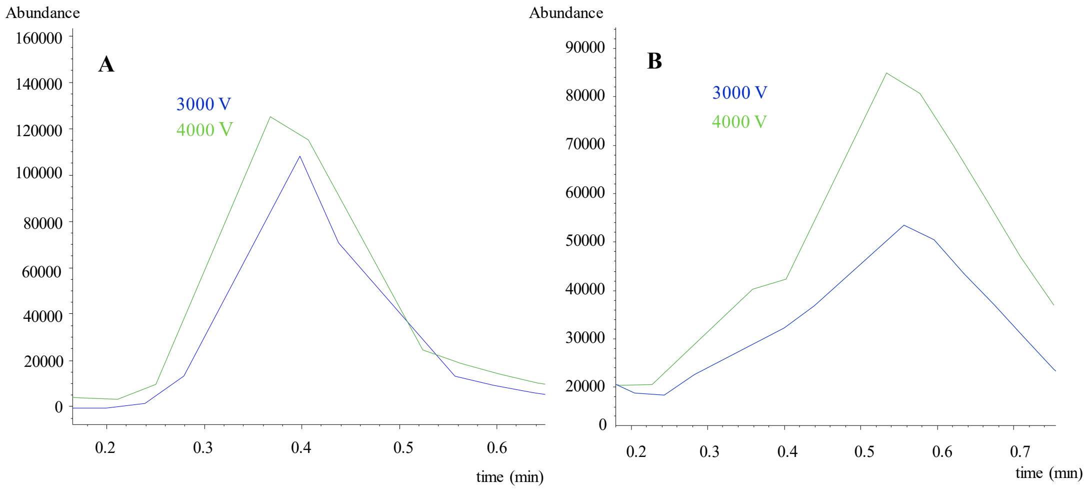

Figure S1. Effect of capillary voltage (V) on the ESI-MS signal intensity of (A) betulin ($[M+H]^+ = 465$) and (B) betulinic acid ($[M\text{-}H]^- = 455$).

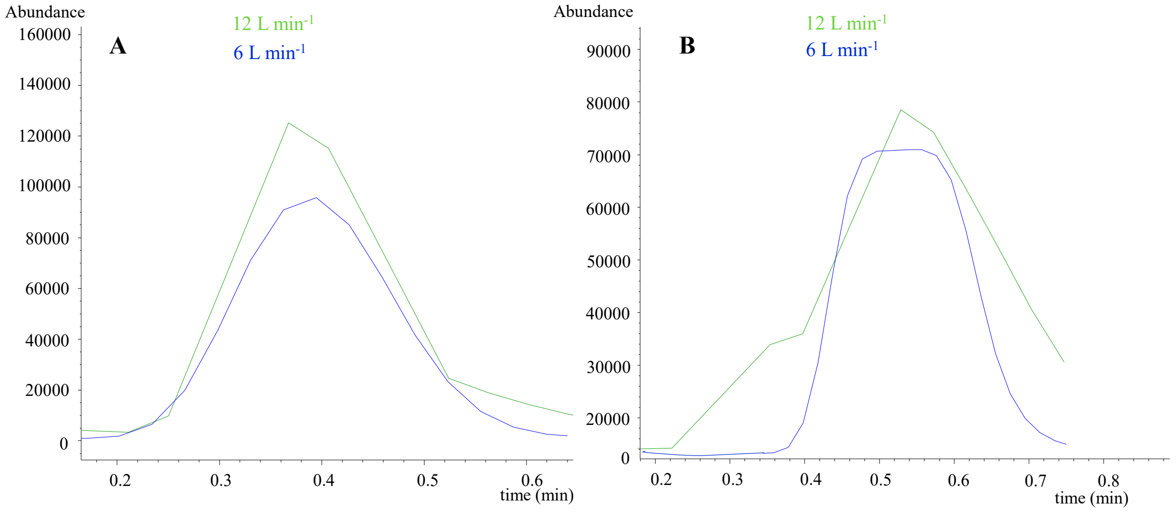

Figure S2: Effect of drying gas flow rate (L min^{-1}) on the ESI-MS signal intensity of (A) betulin ($[M+H]^+ = 465$) and (B) betulinic acid ($[M\text{-}H]^- = 455$).

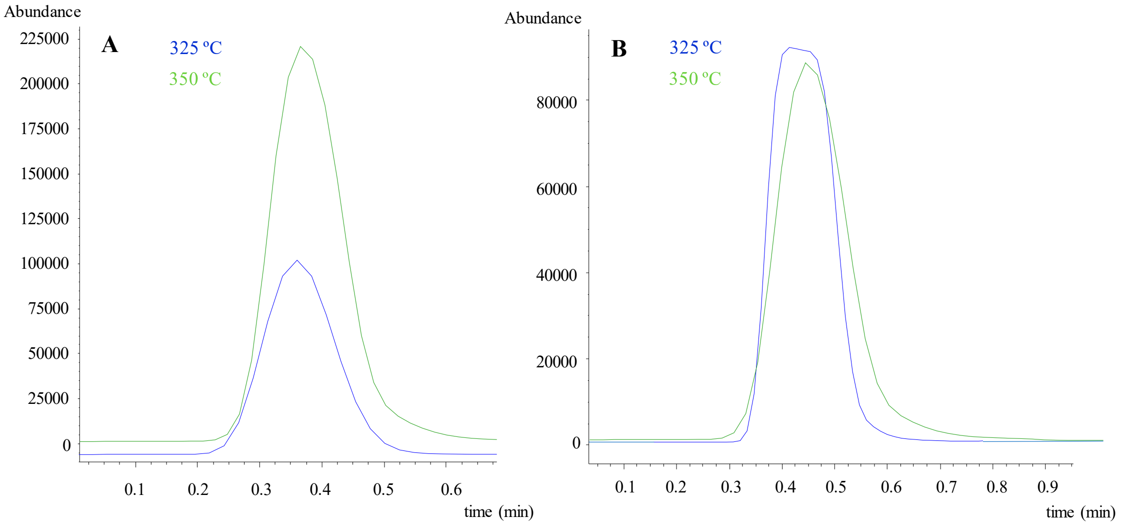

Figure S3: Effect of drying gas temperature (º C) on the ESI-MS signal intensity of (A) betulin ($[M+H]^+ = 465$) and (B) betulinic acid ($[M\text{-}H]^- = 455$).

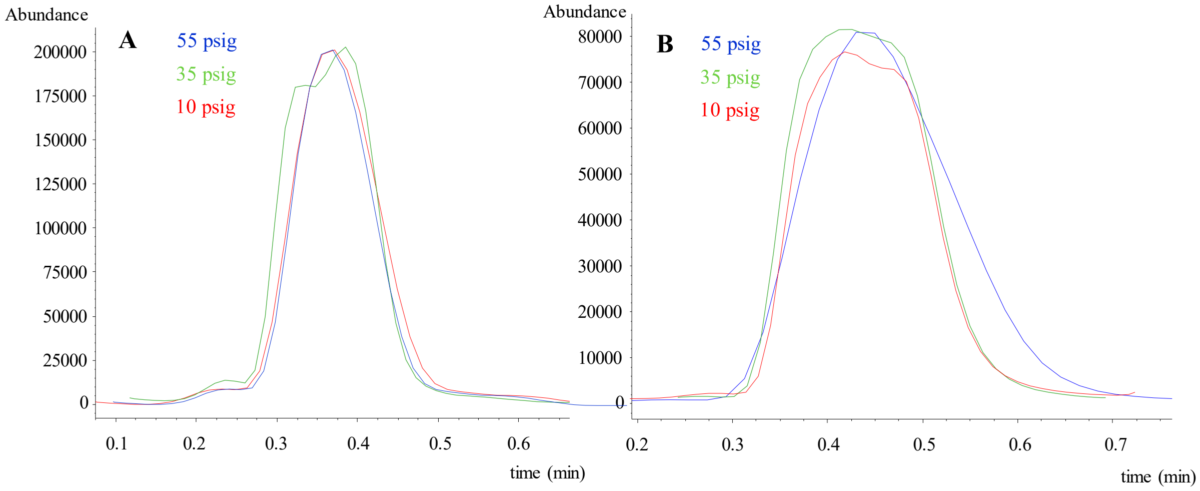

Figure S4: Effect of nebulizing gas pressure (psig) on the ESI-MS signal intensity of (A) betulin ($[M+H]^+ = 465$) and (B) betulinic acid ($[M\text{-}H]^- = 455$).

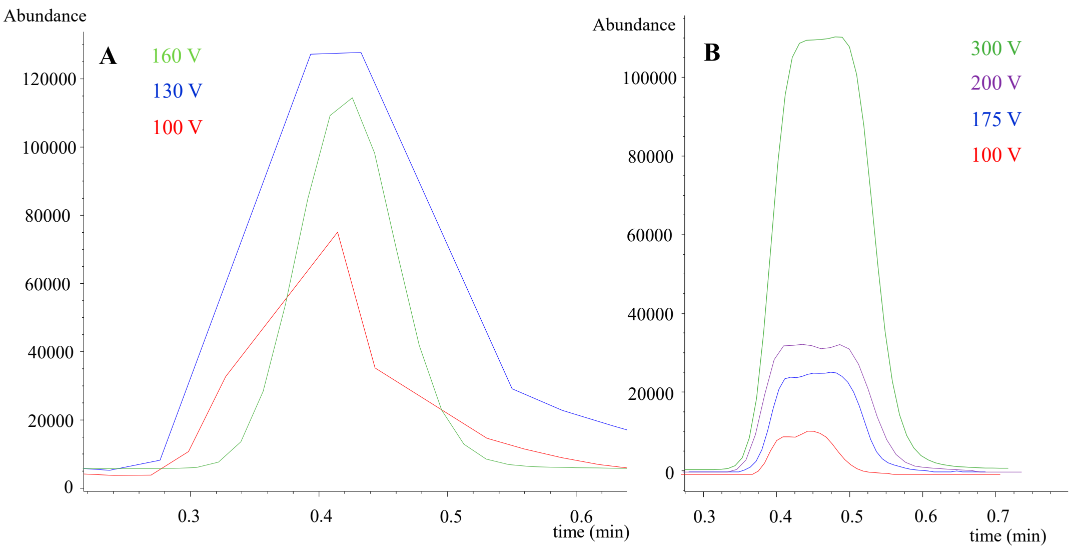

Figure S5: Effect of fragmentor voltage (V) on the ESI-MS signal intensity of (A) betulin ($[M+H]^+ = 465$) and (B) betulinic acid ($[M-H]^- = 455$).
